# Ocular surface tumors

**DOI:** 10.4103/0974-620X.48414

**Published:** 2009

**Authors:** Meghana Anika Varde, Jyotirmay Biswas

**Affiliations:** Medical and Vision Research Foundation, Sankara Nethralaya, Chennai, India

Ocular surface tumors refer mainly to three types of malignant or premalignant neoplasias arising from the conjunctiva and the cornea, i.e., ocular surface squamous neoplasia (OSSN), ocular surface melanocytic tumors and lymphoid tumors of the conjunctiva. Although rare entities, they have a significant impact on patient morbidity and even mortality. A high index of suspicion is needed on the part of the clinician for adequate diagnosis and treatment. The review of ocular surface tumors in this issue of Oman Journal of Ophthalmology comprehensively covers the topic.

Ocular surface squamous neoplasias (OSSN), comprising mainly conjunctival/corneal intraepithelial neoplasm (CIN) including carcinoma in situ and invasive squamous cell carcinoma, occur in about 0.2-12 per 1,000,000 per year.[1] The incidence varies among different geographic locations and ethnic origins. Elderly Caucasian men are more commonly affected, although surveys have shown that the incidence significantly increases with proximity to the equator. Young patients may be affected in the setting of predisposing conditions like xeroderma pigmentosum or immunodeficiency as in HIV, making this entity more common and significant in today’s world. Infection with human papilloma virus (HPV 16 and 18) is associated with malignant OSSN.[2] Ocular surface melanocytic tumors are mainly primary acquired melanosis (PAM) and malignant melanoma. Conjunctival melanomas are very rare. The incidence is estimated at approximately 0.05-0.8 per 1,000,000 per year, with the incidence being higher in persons of Caucasian than of Asian or African origin. The most important etiological factor for the development of acquired melanocytic lesions is ultraviolet light exposure. Conjunctival lymphoma develop in the 5th to 7th decade of life and may arise from B-cells of the conjunctiva-associated lymphatic tissue (CALT) or more seldom as part of systemic disease. The incidence of ocular lymphoma in general has been increasing during the past decades. It nevertheless is a rare tumor. Incidence rates are age-dependent and range between 0.8 and 2.4 per 1,000,000 per year.[3]

In most cases of ocular surface neoplasms, a presumptive clinical diagnosis can be made with a high index of suspicion. Since the benign and precancerous lesions cannot always be easily differentiated clinically, the final diagnosis is made histopathologically. This is particularly so in cases of lymphoproliferations, where differentiation of benign from malignant lesions requires immunohistochemistry to demonstrate monoclonality.

Impression cytology for the establishment of the diagnosis and follow-up has been well demonstrated. Correlation between histopathology and impression cytology could be demonstrated in about 80% of cases of OSSN.[4] The technique is fast to perform, noninvasive and cost-effective. Nevertheless it needs immediate processing of the specimen as well as significant expertise from the pathologist, limiting its application to few selected centers. Impression cytology can be used as a screening technique in the primary assessment and in the follow up of ocular surface neoplasms to detect dysplastic cells. It cannot, however, differentiate between carcinoma in situ and invasive tumors. Cytological changes similar to that of malignancy have been demonstrated following topical mitomycin C (MMC) treatment of PAM. Additionally, the predictive value of impression cytology can be hampered in keratinized surface neoplasms. In such cases, conventional biopsy is recommended.[4]

Management of ocular surface neoplasms depends on the presumptive diagnosis and ranges from serial observation for benign tumors, to incisional or excisional biopsy, cryotherapy, chemotherapy, radiotherapy, enucleation or exenteration. Incisional biopsy should be reserved for extensive tumors that are symptomatic and/or suspected to be malignant. In cases of tumors that involve four clock hours or less of circumference, an excisional biopsy is preferred. Excisional biopsies where malignancy is suspected should be carefully done using a no-touch technique; adjunctive cryotherapy should be applied to the resection margins, and the base should be treated with alcohol and electrocautery. In cases where the tumor seems adherent to the underlying sclera and/or cornea, a partial lamellar sclerokeratoconjunctivectomy as described by Shields is indicated.[5] Wound closure can be done directly or using mucous membrane grafts or amniotic membranes. Cryotherapy alone can be used for the treatment of PAM or pagetoid spreading sebaceous cell carcinoma. Topical chemotherapeutics have the advantage of covering and treating the entire ocular surface, but do not penetrate into the depth; their use is, therefore, limited to flat lesions. Chemotherapeutics commonly used for ocular surface neoplasms are MMC, 5-fluoruracil (5-FU) and interferon alpha-2b (IFN alpha-2b). MMC is a cell-cycle nonspecific alkylating agent and is used for topical treatment of PAM and CIN or as adjunctive therapy after surgical excision of an ocular surface tumor. The topical use is with 0.02% or 0.04% eye drops, applied 4 times a day for 2 weeks and repeated after an interval of 4–6 weeks.[6] 5-FU is a cell-cycle dependent antimetabolite, which inhibits DNA and RNA synthesis and is used in a concentration of 1% in a similar fashion to MMC. Adverse changes in the nasopharyngeal mucosa can occur with both chemotherapeutics, and therefore, the use of punctual plugs is recommended for the duration of the treatment. The topical use of IFN alpha-2b, an immunomodulating agent, has recently been introduced, and promising results have been obtained with complete resolution of conjunctival and corneal intraepithelial neoplasias. Local adverse effects such as discomfort, conjunctival hyperemia and superficial punctate keratopathy, which can be seen in patients treated with topical MMC or 5-FU are also noted with IFN alpha-2b treatment. In addition, corneal epithelial toxicity with formation of persisting microcysts, as known in patients treated with systemic IFN, has been described.[7] If ocular surface lymphomas are confined to the conjunctiva, external beam radiotherapy with 2,000–4,000 Gy is the treatment of choice. Alternative therapies include excisional biopsy and cryotherapy, local interferon injections, or observation. In case of systemic disease, chemotherapy has to be started.[5]
